# HLA-B^*^13:01 as a Risk Allele for Antiepileptic Drugs-Induced Cutaneous Adverse Reactions: Higher Risk for Cross-Reactivity?

**DOI:** 10.3389/fneur.2019.00614

**Published:** 2019-06-11

**Authors:** Fu-Li Min, Bi-Jun Mao, Zhong-Zheng Zheng, Na He, Cui-Xia Fan, Rui-Yan Cai, Juan Wang, Yang-Mei Ou, Bing Qin, Wei-Ping Liao, Yong-Hong Yi, Ze Li, Yi-Wu Shi

**Affiliations:** ^1^Institute of Neuroscience and Department of Neurology of the Second Affiliated Hospital of Guangzhou Medical University, Key Laboratory of Neurogenetics and Channelopathies of Guangdong Province and the Ministry of Education of China, Guangzhou, China; ^2^Department of Neurology, Guangzhou First People's Hospital, School of Medicine, South China University of Technology, Guangzhou Medical University, Guangzhou, China; ^3^Shanghai Tissue Bank Biotechnology Co., Ltd, Shanghai, China; ^4^The First Affiliated Hospital of Xinxiang Medical University, Xinxiang, China; ^5^Guangdong 999 Brain Hospital, Guangzhou, China; ^6^Epilepsy Center and Department of Neurosurgery, The First Affiliated Hospital, Jinan University, Guangzhou, China

**Keywords:** antiepileptic drugs, cross-reactivity, HLA allele, HLA-B^*^13:01, genetic marker

## Abstract

Antiepileptic drugs frequently cause cutaneous adverse reactions (cADRs). Numerous studies have reported associations between human leukocyte antigen (HLA) alleles and cADRs caused by single antiepileptic drug in Southern Han Chinese people. However, the relationship between the HLA allele and cADRs sequentially induced by two or more antiepileptic drugs (AEDs-induced cross-reactivity) is unclear. To explore the associations between HLA alleles and AEDs-induced cross-reactivity, we prospectively recruited patients with AEDs-induced cross-reactivity from 2009 to 2017 and performed high-resolution genotyping to detect the HLA-A, B, C, and DRB1 alleles in patients for comparison with normal controls. To verify the important genotype, we compared its presence in patients with cross-reactivity to enlarged normal controls, and its presence in patients with carbamazepine (CBZ)-induced maculopapular exanthema (MPE) to CBZ-tolerant controls. Further, the important allele was replicated by meta-analysis. Twenty-three patients with AED-induced cross-reactivity and 500 healthy individuals were enrolled from Southern China. All patients had a mild rash without mucosal or systemic involvement. The HLA-B^*^13:01 allele was present in 34.78% (8/23) of patients, 14.60% (73/500) of healthy individuals, and 14.5% (763/5,270) healthy individuals, revealing a significant association (8/23 vs. 73/500; *P* = 0.02; OR: 3.12; 95% CI: 1.28–7.62; 8/23 vs. 763/5,270; *P* = 0.014; OR: 3.15; 95% CI: 1.33–7.46). HLA-B^*^13:01 was presented numerically higher in CBZ-induced MPE than that in CBZ-tolerant individuals without statistical significance (33/145, 22.76%, vs. 28/179, 15.64%; *P* = 0.103). Meta-analysis revealed an association between HLA-B^*^13:01 and cADRs induced by single AEDs or/and non-AEDs in Chinese and Thai populations (*P* = 0.000). This study suggests that HLA-B^*^13:01 is potentially associated with AED-cADRs in general, possibly with stronger effect in cross-reactivity. Screening for HLA-B^*^13:01 prior to starting AEDs therapy may help to avoid cADRs. However, this association requires further analysis in a multi-center study with a larger sample size.

## Introduction

Carbamazepine (CBZ), lamotrigine (LTG), oxcarbazepine (OXC), phenytoin (PHT), and phenobarbital (PB) share a similar aromatic structure and are widely used antiepileptic drugs (AEDs) to control seizures, trigeminal neuralgia, etc. However, they frequently lead to cutaneous adverse reactions (cADRs) (1, 2). cADRs comprise 10–30% of all reported adverse drug reaction (3–5), which vary from mild maculopapular eruption (MPE), hypersensitivity syndrome (HSS), to Stevens-Johnson syndrome (SJS) and/or toxic epidermal necrolysis (TEN) (6). The most severe phenotypes SJS and TEN show high morbidity and mortality (7). It has been reported that 3.61% of patients treated with AEDs experience a skin rash induced by single AED, while 0.48% of patients show cross-reactivity sequentially induced by two or more AEDs (AEDs-induced cross-reactivity) in Han Chinese people, including aromatic and non-aromatic AEDs such as valproic acid, levetiracetam, and topiramate (8). cADRs frequently result in drug withdrawal, which increases the risk of seizure worse and medical costs (9, 10).

Although the mechanism of AEDs-induced cADRs is unclear, numerous studies have suggested that the major histocompatibility complex (also known as human leukocyte antigen, HLA) is involved in cADRs (9). Several HLA alleles were found to be associated with cADRs caused by single drug. The HLA-B^*^15:02 allele is a strong risk factor for CBZ-induced SJS in Han Chinese people (11, 12), HLA-A^*^24:02 is a common risk factor for aromatic AEDs induced SJS in the Southern Han Chinese people (13), and HLA-A^*^31:01 is associated with CBZ-induced cADRs in Japanese and Caucasian people (14, 15). However, the HLA-related genetic risk factors of AEDs-induced cross-reactivity are unclear.

Here, we performed a case-control study to investigate the relationship between AEDs-induced cross-reactivity and HLA alleles in a Southern Han Chinese population.

## Materials and Methods

### Participants

Individuals were enrolled in southern China from 2009 to 2017, and treated at the Second Affiliated Hospital of Guangzhou Medical University, Guangzhou First People's Hospital, the First Affiliated Hospital of Jinan University, and Guangdong 999 Brain Hospital in Guangzhou. The study was approved by the hospital ethics committee. All participants or their parents provided written informed consent in accordance with the Declaration of Helsinki.

Cases with cross-reactivity were defined as sequential rashes in response to two or more AEDs in the same individual. For example, when a patient experienced a skin rash induced by CBZ, CBZ was discontinued, and 2 weeks later the patient was treated with LTG, then rashes appeared again. The diagnosis of AEDs-induced cross-reactivity was confirmed by a dermatologist based on the clinical and morphological characteristics of the patients' skin. MPE was defined as skin eruptions without mucosal or systemic involvement. SJS and TEN were diagnosed according to Roujeau's diagnostic criteria. Hypersensitivity syndrome was defined as a fever, rash, eosinophilia, and systemic manifestation (e.g., hepatitis and nephritis).

Five-hundred healthy volunteers who have not taken AEDs were recruited as normal controls. To verify the significant allele, we further increased the sample size of normal controls from 500 to 5,270. All healthy volunteers were recruited from the Healthy Physical Examination Center in the Second Affiliated Hospital of Guangzhou Medical University, and had not taken AEDs. Because most of the patients had taken CBZ, we recruited another cohort comprising of individuals with CBZ-induced MPE and CBZ-tolerant to clarify whether AEDs-induced cross-reactivity and CBZ-induced MPE have the same risk HLA allele. The cases with CBZ-induced MPE and CBZ tolerant controls were recruited from the Second Affiliated Hospital of Guangzhou Medical University between 2007 and 2018. CBZ tolerant controls were participants with epilepsy who took CBZ for at least 3 months without evidence of cutaneous adverse reactions.

All cases and controls were Southern Han Chinese.

Sequence based high-resolution HLA-A/B/C/DRB1 genotyping was performed by Shanghai Tissue Bank Biotechnology Co., Ltd. (Shanghai, China).

### Statistical Analysis

We performed statistical analyses using SPSS version 17.0 (SPSS, Inc., Chicago, IL, USA). Fisher's exact tests were used to assess the difference in the presence of HLA alleles between the case and control groups. Because CBZ and LTG were the most common culprit drugs, we conducted an independent *t*-test to compare the mean age, latency to cADRs, and initial and maximum dosages between one group of CBZ/LTG as the first culprit drug and the other group of CBZ/LTG as the second culprit drug. *P* < 0.05 (two-sided) were considered significantly different. The corrected *P* (*P*c) values were estimated by Bonferroni's correction for multiple comparisons (*n* = 12, 16, and 14 for HLA-A, HLA -B, and HLA-C alleles, respectively).

### Meta-Analysis

We performed meta-analyses on data obtained from other studies to investigate the relationship between HLA-B^*^13:01 allele and cADRs induced by AEDs or/and non-AEDs. A complete search of online databases, including MEDLINE, EMBASE, Google Scholar, was conducted.

The following terms were used in our searches: “HLA-B^*^13:01” or “human leukocyte antigen B^*^13:01,” “Stevens–Johnson syndrome” or “SJS,” “toxic epidermal necrolysis” or “TEN,” “cutaneous adverse drug reactions” or “cADRs,” “maculopapular eruption” or “MPE,” “Antiepileptic drugs” or “AEDs.” The latest search was conducted on April1, 2018.

Criteria for the selection of studies were: (1) the report was of a case-control study on association between HLA-B^*^13:01 and cADRs induced by AEDs or non-AEDs; (2) the genotyping method and ethnicity were provided; (3) the presence of HLA-B^*^13:01 in the cases, either the population controls or tolerant controls was reported or could be obtained from the authors or other sources; (4) the most recent publication with the largest number of samples was selected when duplicate publications were identified. Exclusion criteria were: (1) reports were not of case-control studies; (2) repeated studies; (3) studies did not indicate the presence of HLA-B^*^13:01 in the case group and control group; (4) abstracts and reviews; (5) non-human studies.

The following information were extracted: the first author of the study, publication year, ethnicity of the study population, presence of HLA-B^*^13:01 allele among cADRs cases and controls, total number of cADRs cases and controls, and main results. The methodological quality was assessed according to the recommendations of the Cochrane Collaboration Handbook (https://www.cochrane.de).

Data managements and analyses were conducted using STATA (Version 10.1 Stata Corp LP, College Station, TX, USA). Odds ratios (ORs) with corresponding 95% confidence intervals (CIs) were calculated to verify the association between the HLA-B^*^13:01 allele and drugs-induced cADRs. Begg's test was used to evaluate publication bias (16). Statistical heterogeneity among studies was assessed via the Q statistic and *I*2 tests (17). A *P* < 0.1 and an *I*^2^-value >50% were defined as evidence of statistical heterogeneity, under which the association was assessed using the random model. Analyses were also performed separately on studies using different types of control patients (e.g., drug-tolerant or normal controls) and different drugs induced cADRs.

Seven articles met the criteria. Data from 8 studies, including the present study, were used for meta-analysis. The data included the populations from Thailand and China.

## Results

### Characteristics of Patients

A total of 23 patients with AEDs-induced cross-reactivity and 500 healthy volunteers were recruited in this study. The demographic variables and clinical manifestations of cases are summarized in Table 1. There were 14 females and 9 males. The average age was 24 years (range 6–53 years). All cases occurred as sequential rashes induced by two of the following drugs: CBZ, LTG, OXC, levetiracetam, topiramate, valproic acid, PB, and PHT. The phenotype of AED-induced cross-reactivity in this cohort was MPE without mucosal or systemic involvement.

**Table 1:** Demographic and clinical characteristics of AEDs-induced cross-reactivity.

**Number**	**Sex**	**Age^**a**^**	**First culprit drug**				**Second culprit drug**			
			**Culprit drug**	**Latency to cADRs(days)**	**Initial dose (mg/day)**	**Maximum dose (mg/day)**	**Culprit drug**	**Latency to cADRs (days)**	**Initial dose (mg/day)**	**Maximum dose (mg/day)**
CR1	F	53	CBZ	15	300	600	LTG	9	12.5	12.5
CR2	F	23	CBZ	7	300	500	LTG	13	12.5	25
CR3	M	30	CBZ	32	500	500	OXC	7	25	25
CR4	F	43	CBZ	5	200	200	OXC	6	150	300
CR5	F	24	CBZ	9	200	400	OXC	16	150	450
CR6	M	34	CBZ	13	200	600	OXC	18	150	450
CR7	F	29	CBZ	17	200	600	OXC	17	150	450
CR8	M	17	CBZ	6	200	200	TPM	21	50	100
CR9	F	7	LTG	2	25	25	CBZ	14	200	400
CR10	F	39	LTG	9	25	50	CBZ	13	600	600
CR11	M	24	LTG	11	12.5	25	CBZ	15	200	400
CR12	M	29	LTG	15	12.5	25	CBZ	16	200	500
CR13	M	6	LTG	16	12.5	25	CBZ	8	200	200
CR14	F	20	LTG	7	25	25	OXC	5	300	600
CR15	F	16	LTG	7	12.5	25	OXC	7	300	300
CR16	F	27	LTG	9	25	25	LEV	8	500	500
CR17	F	7	LTG	20	3.125	12.5	VPA	10	250	250
CR18	M	18	LTG	9	12.5	25	TPM	50	50	50
CR19	M	20	PHT	12	100	200	LTG	9	25	25
CR20	F	37	PHT	16	100	300	OXC	13	300	600
CR21	M	28	OXC	7	300	300	LTG	7	100	100
CR22	F	33	VPA	21	500	500	LTG	16	25	50
CR23	F	7	PB	13	50	100	CBZ	7	200	300

a *Age at development of cross-sensitivity*.

*F, female; M, male; CBZ, carbamazepine; LEV, levetiracetam; LTG, lamotrigine; OXC, oxcarbazepine; PB, phenobarbital; PHT, phenytoin; TPM, topiramate; VPA, sodium valproate*.

CBZ and LTG were the most common culprit drugs. With respect to the sex ratio, mean age, latency to cADRs, and initial and maximum dosages, no significant difference was found between one group of CBZ/LTG as the first culprit drug and the other group of CBZ/LTG as the second culprit drug.

### HLA-B^*^13:01 Is Associated With Antiepileptic Drugs-Induced Cross-Reactivity

The HLA genotypes of patients with AEDs-induced cross-reactivity are shown in Table 2. The distribution of HLA alleles in the 23 cases and 500 normal controls is shown in Table 3. Three alleles were significantly associated with AEDs-induced cross-reactivity. The HLA-B^*^13:01 allele was found in 8 (34.78%) of the 23 AEDs-induced cross-reactivity patients and 73 (14.60%) of the 500 normal controls (*P* = 0.02; OR: 3.12; 95% CI: 1.28–7.62; *Pc* = 0.32, *n* = 16 for HLA-B^*^13:01 correction). The HLA-A^*^11:02 allele was found in two (8.70%) of the 23 AEDs-induced cross-reactivity patients, and in none (0%) of the 500 normal controls (*P* = 0.002; OR: 68.32; 95% CI: 6.83–683.53; *Pc* = 0.02, n = 12 for HLA-A^*^11:02 correction). The HLA-C^*^04:03 allele was found in three (13.64%) of the 22 AED-induced cross-reactivity patients and 10 (2.01%) of the 498 normal controls (*P* = 0.01; OR: 7.71; 95% CI: 1.96-30.3; *Pc* = 0.14, *n* = 14 for HLA-C^*^04:03 correction). Furthermore, one case with HLA-A^*^11:02 and one case with HLA-C^*^04:03 were positive for HLA-B^*^13:01. If excluding these cases, the presence of HLA-A^*^11:02 and C^*^04:03 in the patients was 4.35% (1/23) and 9.09% (2/22), respectively. The presence of the two alleles HLA-A^*^11:02 and C^*^04:03 is very low in the normal Chinese population (http://www.allelefrequencies.net, such as 0 and 2.01% in this cohort). These results suggested HLA-B^*^13:01 should be considered further. To exclude the possibility of lost significance, we compared the presence of HLA-B^*^13:01 between the 23 patients of AEDs-induced cross-reactivity and a larger control cohort containing 5,270 normal individuals, which revealed a significant association between HLA-B^*^13:01 and AEDs-induced cross-reactivity (8/23, 34.78%, vs. 763/5,270, 14.48%; *P* = 0.014, OR:3.15, 95%CI: 1.33–7.46).

**Table 2:** Genotypes in the 23 patients with AEDs-induced cross-reactivity.

**Number**	**HLA-A**	**HLA-B**	**HLA-C**	**HLA-DRB1**	**Culprit drug**
CR1	02:01/33:03	**13:01**/38:02	03:04/07:02	08:03/09:01	CBZ/LTG
CR2	02:03/11:01	**13:01/13:01**	03:04/03:04	12:02/16:02	CBZ/LTG
CR3	11:01/11:01	15:02/15:18	02:02/08:01	04:04/15:01	CBZ/OXC
CR4	11:01/31:01	15:01/56:04	01:02/03:03	14:54/15:02	CBZ/OXC
CR5	11:01/11:01	**13:01**/35:01	03:03/03:04	09:01/15:01	CBZ/OXC
CR6	02:07/02:07	46:01/46:01	01:02/01:02	09:01/09:01	CBZ/OXC
CR7	02:01/11:01	15:25/39:01	**04:03**/07:02	09:01/16:02	CBZ/OXC
CR8	11:01/**11:02**	**13:01**/58:01	03:02/03:04	03:01/15:01	CBZ/TPM
CR9	11:01/24:02	40:01/40:01	03:04/07:02	08:03/14:54	LTG/CBZ
CR10	24:02/24:02	39:01/48:01	07:02/15:02	12:02/15:01	LTG/CBZ
CR11	11:01/02:07	15:02/46:01	01:02/08:01	09:01/09:01	LTG/CBZ
CR12	11:01/74:02	**13:01**/51:01	08:01/14:02	03:01/14:04	LTG/CBZ
CR13	11:01/11:01	40:01/40:01	None	None	LTG/CBZ
CR14	02:01/33:03	40:01/58:01	03:02/15:02	03:01/11:01	LTG/OXC
CR15	02:03/11:01	40:01/40:01	**04:03**/07:66	12:02/15:01	LTG/OXC
CR16	02:03/**11:02**	40:01/40:01	07:02/07:02	15:01/15:02	LTG/LEV
CR17	02:03/24:02	35:01/40:01	03:04/04:01	09:01/14:05	LTG/VPA
CR18	30:01/30:01	13:02/13:02	06:02/06:02	07:01/07:01	LTG/TPM
CR19	02:03/02:07	46:01/38:02	01:02/07:02	04:05/16:02	PTH/LTG
CR20	02:07/23:01	45:01/46:01	01:02/06:02	07:01/08:03	PHT/OXC
CR21	02:07/02:07	**13:01**/46:01	01:02/03:04	07:01/15:01	OXC/LTG
CR22	11:01/11:01	**13:01**/15:25	03:04/**04:03**	12:02/12:02	VPA/LTG
CR23	02:01/24:20	**13:01**/40:01	03:02/07:02	12:02/15:01	PB/CBZ

*AEDs, antiepileptic drugs*.

*Bold black indicates those alleles are significant*.

*None: individual was not subjected to HLA genotyping because of insufficient DNA*.

^*^ Individual was positive for HLA-B^*^13:01 and HLA-A^*^11:02, or positive for HLA-B^*^13:01 and HLA-C^*^04:03.

**Table 3:** Association between HLA alleles and AEDs-induced cross-reactivity.

	**No. of HLA genotypes / Total no. (%)**		**Cases vs. Normal Controls**	
**Allele**	**Cross-reactivity Case (*n* = 23 or 22^**a**^)**	**Normal Controls (*n* = 500 or 498^**a**^)**	***P*-value**	**OR (95% CI)**
A^*^02:01	4/23 (17.39)	74/500 (14.8)	0.97	1.21 (0.40–3.66)
A^*^02:03	5/23 (21.74)	44/500 (8.80)	0.09	2.88 (1.02–8.13)
A^*^02:07	5/23 (21.74)	117/500 (23.40)	0.85	0.91 (0.33–2.50)
A^*^11:01	12/23 (52.17)	252/500 (50.40)	0.87	1.07 (0.47–2.48)
A^*^11:02	2/23 (8.70)	0/500 (0.00)	0.002^*^	68.32 (6.83–683.53)
A^*^23:01	1/23 (4.35)	1/500 (0.20)	0.09	22.68 (1.37–374.68)
A^*^24:02	3/23 (13.04)	148/500 (29.60)	0.09	0.36 (0.10–1.22)
A^*^24:20	1/23 (4.35)	3/500 (0.60)	0.17	7.53 (0.75–75.34)
A^*^30:01	1/23 (4.35)	24/500 (4.80)	1.00	0.90 (0.12–6.97)
A^*^31:01	1/23 (4.35)	21/500 (4.20)	1.00	1.04 (0.13–8.06)
A^*^33:03	2/23 (8.70)	59/500 (11.80)	0.90	0.71 (0.16–3.11)
A^*^74:02	1/23 (4.35)	0/500 (0.00)	0.04	43.57 (3.81–498.11)
B^*^13:01	**8/23 (34.78)**	**73/500 (14.60)**	0.02^*^	**3.12 (1.28–7.62)**
B^*^13:02	1/23 (4.35)	29/500 (5.80)	1.00	0.74 (0.10–5.67)
B^*^15:01	1/23 (4.35)	35/500 (7.00)	0.94	0.60 (0.08–4.61)
B^*^15:02	2/23 (8.70)	64/500 (12.80)	0.80	0.65 (0.15–2.83)
B^*^15:18	1/23 (4.35)	5/500 (1.00)	0.24	4.50 (0.50–40.17)
B^*^15:25	2/23 (8.70)	12/500 (2.40)	0.12	3.87 (0.81–18.42)
B^*^35:01	2/23 (8.70)	25/500 (5.00)	0.76	1.81 (0.40–8.15)
B^*^38:02	2/23 (8.70)	33/500 (6.60)	1.00	1.35 (0.30–6.00)
B^*^39:01	2/23 (8.70)	18/500 (3.60)	0.22	2.55 (0.56–11.72)
B^*^40:01	7/23 (30.43)	178/500 (35.60)	0.61	0.79 (0.32–1.96)
B^*^45:01	1/23 (4.35)	2/500 (0.40)	0.13	11.32 (0.99–129.61)
B^*^46:01	5/23 (21.74)	136/500 (27.20)	0.56	0.74 (0.27–2.04)
B^*^48:01	1/23 (4.35)	7/500 (1.40)	0.30	3.20 (0.38–27.17)
B^*^51:01	1/23 (4.35)	38/500 (7.60)	0.86	0.55 (0.07–4.21)
B^*^56:04	1/23 (4.35)	1/500 (0.20)	0.09	22.68 (1.37–374.68)
B^*^58:01	1/23 (4.35)	51/500 (10.20)	0.58	0.40 (0.05–3.03)
C^*^01:02	6/22 (27.27)	166/498 (33.33)	0.55	0.75 (0.29–1.95)
C^*^02:02	1/22 (4.55)	0/498 (0.00)	0.04	45.36 (3.96–519.49)
C^*^03:02	3/22 (13.64)	86/498 (17.27)	0.88	0.76 (0.22–2.61)
C^*^03:03	2/22 (9.09)	50/498 (10.04)	1.00	0.90 (0.20–3.95)
C^*^03:04	8/22 (36.36)	110/498 (22.09)	0.19	2.02 (0.82–4.93)
C^*^04:01	1/22 (4.55)	20/498 (0.42)	0.60	1.14 (0.15–8.89)
C^*^04:03	3/22 (13.64)	10/498 (2.01)	0.01^*^	7.71 (1.96–30.30)
C^*^06:02	2/22 (9.09)	40/498 (8.03)	1.00	1.15 (0.26–5.08)
C^*^07:02	7/22 (31.82)	156/498 (31.33)	0.96	1.02 (0.41–2.56)
C^*^07:66	1/22 (4.55)	0/498 (0.00)	0.04	45.36 (3.96–519.49)
C^*^08:01	3/22 (13.64)	102/498 (20.48)	0.61	0.61 (0.18–2.11)
C^*^14:02	1/22 (4.55)	38/498 (7.63)	0.90	0.58 (0.08–4.40)
C^*^15:02	2/22 (9.09)	26/498 (5.22)	0.76	1.82 (0.40–8.19)
DRB1^*^03:01	3/22 (13.64)	39/500 (7.80)	0.56	1.87 (0.53–6.59)
DRB1^*^04:04	1/22 (4.55)	5/500 (1.00)	0.23	4.71 (0.53–42.17)
DRB1^*^04:05	1/22 (4.55)	38/500 (7.60)	0.91	0.58 (0.08–4.42)
DRB1^*^07:01	3/22 (13.64)	43/500 (8.60)	0.67	1.68 (0.48–5.90)
DRB1^*^08:03	3/22 (13.64)	101/500 (20.20)	0.63	0.62 (0.18–2.15)
DRB1^*^09:01	6/22 (27.27)	175/500 (35.00)	0.46	0.70 (0.27–1.81)
DRB1^*^11:01	1/22 (4.55)	59/500 (11.80)	0.48	0.36 (0.05–2.70)
DRB1^*^12:02	5/22 (22.73)	100/500 (20.00)	0.97	1.18 (0.42–3.27)
DRB1^*^14:04	1/22 (4.55)	4/500 (0.80)	0.19	5.91 (0.63–55.16)
DRB1^*^14:05	1/22 (4.55)	17/500 (3.40)	0.55	1.35 (0.17–10.65)
DRB1^*^14:54	2/22 (9.09)	40/500 (8.00)	1.00	1.15 (0.26–5.10)
DRB1^*^15:01	8/22 (36.36)	119/500 (23.80)	0.18	1.83 (0.75–4.47)
DRB1^*^15:02	2/22 (9.09)	21/500 (4.20)	0.25	2.28 (0.50–10.41)
DRB1^*^16:02	3/22 (13.64)	44/500 (8.80)	0.69	1.64 (0.47–5.75)

*AEDs, antiepileptic drugs; CBZ, carbamazepine; CI, confidence interval; HLA, human leukocyte antigen; OR, odds ratio*.

*Bold values indicates the allele is an important genotype*.

a *Several individuals were not subjected to HLA genotyping because of insufficient DNA*.

* *P-value is significant and unadjusted*.

Since 18 subjects (18/23, 78.26%) showed cross-reactivity induced by two aromatic AEDs, we compared the presence of HLA-B^*^13:01 between the 18 patients of aromatic AEDs-induced cross-reactivity (6/18, 33.33%) and the two normal control cohorts, which showed no significant difference (6/18 vs. 73/500, *P* = 0.066; 6/18 vs. 763/5,270, *P* = 0.054, respectively).

Because most of the AEDs-induced cross-reactivity patients (14/23, 60.9%) had taken CBZ, we recruited another cohort comprising of individuals with CBZ-induced MPE (145) and CBZ-tolerant controls (179) to clarify whether AEDs-induced cross-reactivity and CBZ-induced MPE have the same risk HLA allele. The HLA-B^*^13:01 presence was significantly higher in AEDs-induced cross-reactivity individuals than in CBZ-tolerant individuals (8/23, 34.78%, vs. 28/179, 15.64%; *P* = 0.049, OR: 2.88, 95%CI: 1.11–7.42), while its presence in CBZ-induced MPE was numerically higher than that in CBZ-tolerant individuals without statistical significance (33/145, 22.76%, vs. 28/179, 15.64%; *P* = 0.103) (Supplementary Table 1).

In addition, in this cohort, two of 23 AEDs-induced cross-reactivity patients (2/23) were positive for HLA-B^*^15:02, three of 23 patients (3/23) were positive for HLA-A^*^24:02, and one patient (1/23) was positive for HLA-A^*^31:01. Compared to the normal controls, there was no significant difference in the presence of HLA-B^*^15:02, HLA-A^*^24:02, or HLA-A^*^31:01 (8.7% vs. 12.80%, *P* = 0.80; 13.04% vs. 29.60%, *P* = 0.09; 4.35% vs. 4.20%, *P* = 1.0, respectively) (Table 3).

### Meta-Analysis: HLA-B^*^13:01 and cADRs

**Figure 1:**
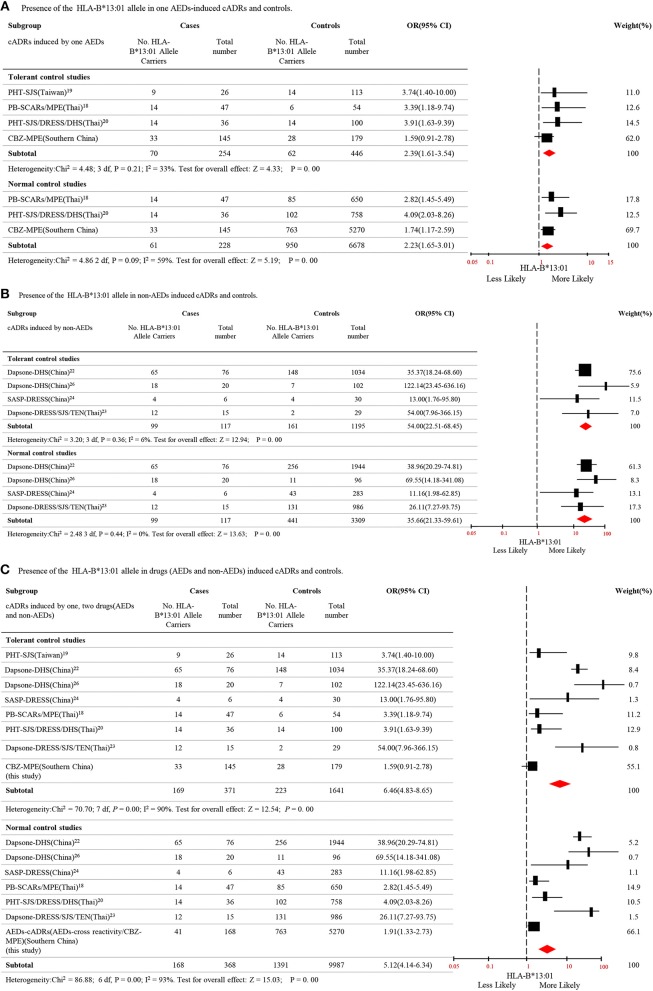
Distribution of HLA-B^*^13:01 allele in cutaneous adverse reactions induced by AEDs and non-AEDs. **(A)** Presence of the HLA-B^*^ 13:01 allele in one AEDs-induced cADRs and controls. **(B)** Presence of the HLA-B^*^ 13:01 allele in non-AEDs induced cADRs and controls. **(C)** Presence of the HLA-B^*^ 13:01 allele in drugs (AEDs and non-AEDs) induced cADRs and controls.

To clarify the relationship between HLA-B^*^13:01 and cADRs, a meta-analysis was performed using data from different populations. Seven case-control studies including Thailand and China population met the criteria (Supplementary Table 2). The meta-analysis analyzed 8 studies, including the present study. The data covered two aspects, i.e., HLA-B^*^13:01 is associated with cADRs induced by single AED, and HLA-B^*^13:01 is associated with cADRs induced by single non-AED. There was a total of 254 cases with cADRs induced by single AED, 446 AEDs tolerant controls, and 6,678 normal controls, and there was a significant association between HLA-B^*^13:01 and cADRs induced by single AED (*P* = 0.000), compared to tolerant controls and normal controls (Figure 1A). There was a total of 117 cases with cADRs induced by single non-AED, 1,195 non-AEDs tolerant controls, and 3,309 normal controls, and there was a significant association between HLA-B^*^13:01 and cADRs induced by single non-AED (*P* = 0.000), compared to tolerant controls and normal controls (Figure 1B). We further combined the cases of cADRs induced by AEDs with the case of cADRs induced by non-AEDs, and there was a significant association between HLA-B^*^13:01 and cADRs in Thai and Chinese populations (*P* = 0.000), compared to tolerant controls and normal controls (Figure 1C).

## Discussion

In the present study, we found that the HLA-B^*^13:01 allele was significantly associated with AEDs-induced cross-reactivity with a sensitivity of 34.78% and specificity of 85.40%. If Bonferroni's correction was performed for multiple comparisons, the significance of HLA-B^*^13:01 would be lost [*Pc* = 0.32 (16 × 0.02)]. Considering the HLA-B was an independent variable, while the frequency of each allele was not. To exclude the possibility of lost significance, we compared the presence of HLA-B^*^13:01 between the 23 patients with AEDs-induced cross-reactivity and a larger control cohort containing 5,270 normal individuals. A significant difference was found between the two groups, suggesting a significant association between HLA-B^*^13:01 and AEDs-induced cross-reactivity. Considering most of the patients (14/23, 60.9%) had taken CBZ, we recruited another cohort comprised of individuals with CBZ-induced MPE (145) and CBZ-tolerant (179) to clarify whether AEDs-induced cross-reactivity and CBZ-induced MPE have the same risk HLA allele. We found that HLA-B^*^13:01 presence was significantly higher in AEDs-induced cross-reactivity individuals than in CBZ-tolerant individuals, while its presence in CBZ-induced MPE was numerically higher than that in CBZ-tolerant control without reaching statistical significance. These results suggest that HLA-B^*^13:01 is a relatively higher risk factor for AEDs-induced cross-reactivity.

Previous studies demonstrated that HLA-B^*^13:01 is associated with PHT-SJS/TEN in Han Chinese people, and with severe cADRs induced by PHT or PB in Thai people (18–21). Meta-analysis of data from independent studies including studies conducted in Thailand and China revealed that HLA-B^*^13:01 may be a potential risk factor for cADRs induced by AEDs (Figure 1A). Additionally, the association with cADRs was not limited to AEDs, and studies reported that HLA-B^*^13:01 was associated with cADRs induced by dapsone and salazosulfapyridine (22–26). Thus, we conducted a meta-analysis to confirm that HLA-B^*^13:01 was associated with cADRs induced by non-AEDs in Chinese and Thai populations (Figure 1B). We further demonstrated that HLA-B^*^13:01 was associated with cADRs induced by drugs including AEDs and non-AEDs (Figure 1C). The HLA-B^*^13:01 allele is present as a medium common allele in Asian populations (https://www.allelefrequencies.net). Based on these data, HLA-B^*^13:01 may be a common genetic marker for predicting cADRs in Asian populations.

cADRs were considered as idiosyncratic immune responses involving the T cell-mediated drug hypersensitivities. At present, the mechanism underlying the HLA involved in AEDs-induced cADRs was unclear, and even for AEDs-induced cross-sensitivity. Previous studies demonstrated that abacavir can bind within the F pocket of the peptide-binding groove of HLA-B^*^57:01 and then alter the repertoire of self-peptides presented to T cells, resulted in abacavir-induced cADRs (27, 28). Specific T-cell receptor is crucial for CBZ-induced SJS in individuals with HLA-B^*^15:02 (29). Symptoms are relieved upon cessation of culprit drug administration, which is consistent with removal of the antigen; however, reintroduction of the drug or a drug with a similar structure can induce an allergic reaction, presumably by rapidly activating memory T cells (30–32). Several studies demonstrated that certain HLAgenotypes can induce T-cell activation in response to a specific drug, resulting in an immune response. The function of the classical HLA molecules is to present the drug for immune surveillance, followed by activation and clonal expansion of CD8^+^T or CD4^+^T cells to trigger immunological responses (33). Activated CD8^+^T or CD4^+^T cells have been detected in the epidermis and dermis of patients with cADRs (34). Further studies are required to explore the mechanism of HLA-B^*^13:01 involvement in AEDs-induced cross-sensitivity.

Additionally, HLA-A^*^11:02 and HLA-C^*^04:03 showed significant association with AEDs-induced cross reactivity. However, one of two cases carrying HLA-A^*^11:02 was positive for HLA-B^*^13:01 and one of three cases carrying HLA-C^*^04:03 was positive for HLA-B^*^13:01. After excluding cases that were also positive for the HLA-B^*^13:01, there was only one patient positive for HLA-A^*^11:02 and two patients positive for HLA-C^*^04:03. HLA-A^*^11:02 and HLA-C^*^04:03 are rare alleles and their frequency were low to 0% in Han Chinese populations (https://www.allelefrequencies.net). Hence, the relationship between HLA-A^*^11:02 or HLA-C^*^04:03 and AEDs-induced cross-reactivity requires further analysis.

Previous studies demonstrated that HLA-B^*^15:02 is specifically associated with CBZ-induced SJS/TEN, HLA-A^*^24:02 is commonly associated with AEDs-induced SJS/TEN in the Han Chinese (13, 35), HLA-A^*^24:02 may be associated with the cross-reactivity of DRESS/MPE induced by PHT and LTG, and HLA-B^*^38:01 may be a major responsible allele for the cross-reactivity of SJS/TEN induced by PHT and LTG in Spanish people (36). However, we failed to find that these alleles were associated with AEDs-induced cross reactivity, the phenotype of the cross-reactivity in this cohort was MPE, which may explain this result. Additionally, a previous study reported an association between the specific haplotype HLA-A^*^02:01/-B^*^35:01/-C^*^04:01 and LTG-induced MPE in Mexican Mestizo people (37), indicating that further studies should explore the relationship between the specific HLA haplotype and cross-reactivity induced by AEDs.

The rate of cross-reactivity caused by two aromatic AEDs was higher than that caused by one aromatic drug and one non-aromatic drug. It was reported that cross-sensitivity induced by aromatic AEDs (CBZ, LTG, OXC, PHT, and PB) occurred in 40–58% of patients because of their similar chemical structures and intermediary metabolism molecules (38). If detoxification of this toxic metabolite is insufficient, it may bind to cellular macromolecules, causing cell necrosis or a secondary immunological response. Among the aromatic drugs, CBZ and OXC, as well as LTG and CBZ, were administered more frequently than the other drugs. Thus, clinicians should use caution when determining replacement drug-therapy among aromatic AEDs; particularly, replace CBZ with OXC, and LTG with CBZ.

We found no significant difference in terms of the sex ratio, mean age, latency to cADRs, and initial and maximum dosages between CBZ/LTG as the first culprit drug and CBZ/LTG as the second culprit drug. Because of the different chemical and pharmacological properties of the causative drug, and heterogeneity of the clinical presentations, it is not surprising that idiosyncratic reactions involve a broad range of mechanism, and that more than one risk alleles may be involved in any single event.

### Further Improvement

This study had several limitations. First, we did not divide the cases into subgroups according to cross-reactivity induced by the same order of drug prescribed due to the limit sample. Second, we did not exactly match the drug tolerant subgroup. To decrease deviation, we investigated the significance of the association by comparison with a large number of normal individuals. A further study with a larger samples size and cases from multiple centers along with exactly matched tolerant controls should be conducted to confirm that the HLA-B^*^13:01 allele is a risk factor for AEDs-induced cross-reactivity, and a common risk factor for cADRs.

## Conclusion

The HLA-B^*^13:01 allele is potentially associated with AED-cADRs in general, possibly with stronger effect in cross-reactivity. Our finding suggests that screening for HLA-B^*^13:01 prior to starting AEDs therapy may help to avoid AEDs-induced cross-reactivity. HLA-A^*^31:01 testing can alert clinicians patients to patients who are at an increased risk of AEDs-induced cross-reactivity, and patients who have already experienced cADRs can be advised to avoid structurally related drugs.

## Ethics Statement

The study was approved by the Second Affiliated Hospital of Guangzhou Medical University ethics committee. All participants or their parents gave written informed consent in accordance with the Declaration of Helsinki.

## Author Contributions

F-LM and B-JM contributed equally to this work. ZL and Y-WS conceived and designed the experiments. F-LM, B-JM, JW, and Z-ZZ performed the experiments. F-LM, B-JM, BQ, and Y-WS analyzed the data. F-LM, B-JM, BQ, Y-MO, JW, Y-HY, W-PL, C-XF, NH, and R-YC collected samples and clinical data. F-LM, B-JM, and Y-WS wrote the paper. All authors have been involved in the study and have approved the final paper.

### Conflict of Interest Statement

Z-ZZ was employed by Shanghai Tissue Bank Biotechnology Co. Ltd, China. The remaining authors declare that the research was conducted in the absence of any commercial or financial relationships that could be construed as a potential conflict of interest. The reviewer PK declared a past co-authorship with several of the authors, BQ, JW, and NH, to the handling editor.

## Abbreviations

CBZ: carbamazepine
LTG: lamotrigine
OXC: oxcarbazepine
PHT: phenytoin
PB: phenobarbital
AED: antiepileptic drug
cADRs: cutaneous adverse reactions
MPE: maculopapular exanthema
HSS: hypersensitivity syndrome
SJS: Stevens-Johnson syndrome
TEN: toxic epidermal necrolysis
VPA: valproic acid
LEV: levetiracetam
TPM: topiramate
HLA: human leukocyte antigen.
