# Supplementary material for: HLA-B*13:01 as a Risk Allele for Antiepileptic Drugs-Induced Cutaneous Adverse Reactions: Higher Risk for Cross-Reactivity?
Source: Front Neurol. 2019 Jun 11;10:614. doi: 10.3389/fneur.2019.00614 (PMC6584797; doi:10.3389/fneur.2019.00614)
Supplement: Supplementary file 1 [file Data_Sheet_1.docx]

Supplementary table 1. The presence of HLA-B*13:01 in CBZ-induced MPE and CBZ-tolerant controls.

|  |  |  | MPE vs. Tolerant controls | | |  |  |
| --- | --- | --- | --- | --- | --- | --- | --- |
| allele | MPE (%) | Tolerant controls (%) | | OR (95%CI) | *P*-value | |  |
| B*13:01 | 33/145(22.76) | 28/179 (15.64) | | 1.59 (0.91-2.78) | 0.103 | | |

Abbreviations: CBZ, carbamazepine; MPE, maculopapular exanthema;

CI, confidence interval; HLA, human leukocyte antigen; OR, odds ratio;

Supplementary table 2. Characteristics of included in the meta-analysis.

| References | Population | | Phenotypes of cADRs | Case/control | Patient on drug  (n) | cADRs patients (%) | Drug tolerant patients  (n) | Normal controls  (n) | Method of genotyping | Reported HLA alleles included in meta-analysis | Comparable group |
| --- | --- | --- | --- | --- | --- | --- | --- | --- | --- | --- | --- |
| Hung et al.  (2010) | | Han Chinese | SJS/TEN | Case: Patients who developed SJS/TEN within 2 months after receiving PHT and the symptoms resolved upon withdrawal of the drug.  Control:Patients who had been on PHT for more than 3 months did not show any cADRs. | 139 | 26 (18.7) | 113 | N | PCR-SSOP  /PCR-SBT | HLA-B*13:01 | SJS/TEN vs. tolerant |
| Wang et al.  (2013) | | Chinese | DIHR | Case: Patients who developed cADRswithin 2 months after receiving dapsonand the symptoms resolved upon withdrawal of the drug.  Control:Patients who had been defined as leprosy with more than 8 weeks of exposure to dapsone but without any episode of drug eruption and reaction. | 122 | 20 (16.4) | 102 | 96 | PCR-SSOP | HLA-B*13:01 | DIHR vs. tolerant DIHRvs. normal  controls |
| Zhang et al.  (2013) | | Chinese | DHS | Case: Patients who developed cADRswithin 2 months after receiving dapsonand the symptoms resolved upon withdrawal of the drug.  Control: who had been cured of leprosy after treatment with dapsone as part of multidrug therapy for at least 6 months but whose status with respect to the dapsone hypersensitivity syndrome could not be determined owing to insufficient medical information. | 1110 | 76 (6.8) | 1034 | 1944 | PCR-SSOP | HLA-B*13:01 | DHS vs. tolerant DHS vs. normal  controls |
| Yang et al.  (2014) | | Han Chinese | DRESS | Case: Patients who developed DRESS after receiving SASP within the frist 2 months of exposure andthe symptoms resolved uponwithdrawal of the drug.  Control:patients who did not develop any cutaneous manifestations during the treatment with SASP for more than  3 months. | 36 | 6 (16.7) | 30 | 283 | PCR-SSOP | HLA-B*13:01 | DRESS vs. tolerant DRESS vs. normal  controls |
| Manuyakorn et al.  (2016) | | Thai | SCARs/MPE | Case: Thai children, between 0 and 18 years of age, who were diagnosed with phenobarbital hypersensitivity, which included SCARs andMPs.  Control: patients were Thai children of a corresponding age who had taken phenobarbital for at least 12 weekswithout anyhypersensitivity  reaction. | 101 | 47 (46.5) | 54 | 650 | PCR-SSOP | HLA-B*13:01 | SCARs/MPE vs. tolerant SCARs/MPE vs. normal  controls |
| Yampayon et al.  (2017) | | Thai | SJS/DRESS/DHS | Cases: Thai adult patients who had PHT-induced SCARs;  Control:patient who had taken PHT for at least 3 monthswith no PHT- cADRs. | 136 | 36 (26.5) | 100 | 758 | PCR-SSOP | HLA-B*13:01 | SJS/DRESS/DHS vs. tolerant SJS/DRESS/DHS vs. normal  controls |
| Tempark et al.  (2017) | | Thai | DRESS/SJS/TEN | Case:Patients who developed SCARs between 4 and 6 weeks after initiation of treatment by using dapsone.  Control:The nonleprosy patients who had been taking dapsone more than 6 months without evidence of cutaneous adverse effects. | 44 | 15 (34.1) | 29 | 986 | PCR-SSOP | HLA-B*13:01 | DRESS/SJS/TEN vs. tolerant DRESS/SJS/TEN vs. normal  controls |

Abbreviations: cADRs, cutaneous adverse drug reaction; SJS, Stevens-Johnson syndrome; TEN, toxic epidermal necrolysis; PHT, phenytoin; HLA, human leukocyte antigen; DIHR, dapsone-induced hypersensitivity reactions; DHS, dapsone hypersensitivity syndrome; DRESS, drug rash with eosinophilia and systemic symptoms; SASP, salazosulfapyridine; SCARs,severe cutaneous adverse drug reactions; MPE, maculopapular exanthema.
